# Proteome profiling of endogenous and potential S-nitrosylation in colorectal cancer

**DOI:** 10.3389/fendo.2023.1153719

**Published:** 2023-04-14

**Authors:** Feng Liang, Shuang Wang, Yu Guo, Yu Mu, FengJia Shang, Min Wang

**Affiliations:** ^1^ Department of General Surgery, The Second Hospital of Jilin University, Changchun, China; ^2^ Department of Dermatology, The Second Hospital of Jilin University, Changchun, China

**Keywords:** colorectal cancer, S-nitrosylation, proteomics, biotin switch, tumor endocrine and metabolic pathways

## Abstract

**Background:**

As a common cancer with high incidence rate and mortality, colorectal cancer (CRC) is seriously threatening human health. S-nitrosylation (SNO) proteins mediated by nitric oxide (NO) has important implications in the genesis, progression, and apoptosis of CRC. It’s worth noting that the SNO proteins also play an important role in the tumor endocrine and metabolic pathways of CRC.

**Materials and methods:**

In this study, the protein extracts of human tissues and cell lines were treated by biotin switch technology and magnetic beads enrichment. The proteomic results of endogenous and potential SNO proteins were analyzed by mass spectrometry (MS). Through the comparison and analysis of MS results, Gene Ontology (GO) analysis, and literatures, some endogenous and potential SNO proteins were identified in CRC, which were closely related to the tumor endocrine and metabolic pathways, the apoptotic signaling pathways, protein maturation, and other biological processes of the proliferation and apoptosis of CRC cells.

**Results:**

A total of 19 proteins containing potential or endogenous SNO sites were detected in both human cancer tissue and SW 480 cells. Through the cross validation of MS results, GO analysis, and literatures, several SNO proteins were identified frequently in CRC, such as the actin, cytoplasmic 1 (ACTB), peroxiredoxin-4 (PRDX4), protein S100A8 (S100A8), pyruvate kinase PKM (PKM), glyceraldehyde-3-phosphate dehydrogenase (GAPDH), which were closely related to the tumor endocrine and metabolic pathways and the apoptotic signaling pathways of CRC.

**Conclusion:**

Different CRC cells and tissues contained potential and endogenous SNO modified proteins. In addition, some SNO proteins could participate in the proliferation, metastasis and apoptosis of CRC by regulating the tumor endocrine and metabolic pathways.

## 1 Introduction

Worldwide, colorectal cancer (CRC) is the third most lethal malignant tumor and the fourth most frequently diagnosed malignant tumor (1, 2). The global incidence and mortality of CRC are increasing because of the continuously expanding and aging of the population with no differences between the sexes (3). CRC accounts for 13–14% of clinical cancer cases and is responsible for 10–13% of total cancer mortality. Therefore, CRC is one of the most epidemic cancers in the world (4). If CRC can be diagnosed early, the tumor is limited and the cure rate after surgery is very high. By contrast, if CRC has metastasized, both the curation and survival rates are very low (3). Surgery is the basis for the treatment of CRC. The tumor, lymph node, and metastasis (TNM) staging system is still the gold standard to predict the prognosis of CRC (5). However, tumors with similar histopathological features might exhibit different clinical outcomes and treatment responses. Therefore, further understanding the biological processes of CRC cells could help the medical community find new methods for the early diagnosis and treatment of CRC (5). Endocrine and metabolism-related biological processes play an important role in the biology of CRC. Currently, research on endocrinology and metabolism of CRC mainly involves insulin resistance, insulin-like growth factor (6), prostaglandin synthesis (7), acute and chronic inflammation (8), and glucose metabolism (9, 10). The proliferation and metastasis of tumor cells are often closely related to the intake and utilization of synthetic materials, and the generation and release of energy. Therefore, the study of the tumor endocrine and metabolic pathways is particularly important to understand tumor development, proliferation, and metastasis.

Post-translational modifications might change the physical and chemical properties of proteins, thus altering their functions (11). Rapid and reversible modifications, such as phosphorylation, palmitoylation, glycosylation, and S-nitrosylation (SNO), are of great significance in the regulation of inflammation and metabolism (11). SNO is an endogenous post-translational modification induced by nitric oxide (NO), and SNO of tumor related proteins might affect the proliferation (12), migration (13), apoptosis (14), and other mechanisms of CRC. SNO refers to the formation of nitroso moieties in cysteine residues, and SNO is also an oxidation reaction involving NO and cysteine mercaptan (15). At the same time, SNO is an important step in the physiological signaling mediated by NO and endogenous nitro-mercaptan (16). A variety of NO derivatives can effectively mediate the nitration of proteins *in vivo* (17). To date, SNO proteins have been proven to be involved in various cellular functions, including structural proteins, ion channels, signal molecule receptors, and enzymes (18). Both low and high expressions of SNO have been shown to be associated with diseases (16). Importantly, SNO modification has all the typical characteristics of signal transduction molecules. SNO is reversible, and concentration and time dependent, and is necessary for specific cell responses (18).

Recently, evidence has emerged that the stability and function of some cancer-related proteins change after SNO modification. For instance, Leon-Bollotte and co-workers incubated colon and mammary cancer cell lines with the NO donor glyceryl trinitrate using the biotin switch assay to monitor SNO of the Fas receptor and Fas mutants to investigate the involvement of SNO in Fas-mediated cell death (14). The study demonstrated that both exogenous and endogenous NO could trigger the SNO of the Fas receptor, which promoted the formation of death inducing signal complexes and induced cell death (14). Another study by Williams and colleagues reported that a high concentration of NO could affect the SNO of β-catenin, the p65 subunit in the nuclear factor kappa B (NF-κB) pathway, and p53 (12). A recent study by Li and co-workers reported that a high concentration of NO could induce SNO of glyceraldehyde-3-phosphate dehydrogenase (GAPDH) in SW 480 cells, which could lead to nuclear translocation after Siah E3 ubiquitin protein ligase 1 (Siah1) binding, thus inducing apoptosis (19). Another study by Wang and colleagues reported that the changes in SNO detected using proteomic analysis were regulated by the expression level of the target proteins [such as thioredoxin and annexin A4 (ANXA4)] (20). In addition, the SNO protein signaling network might be involved in the progression of CRC (20). A study by Helmer and co-workers demonstrated that the deletion of the helicase-like transcription factor in the tumor microenvironment of CRC could lead to the trans activation of inducible nitric oxide synthase (iNOS), leading to the reprogramming of SNO sites of tumor proteins in the iNOS-S100 calcium binding protein (S100A8/A9) signaling axis. These changes promoted inflammation and tumor metastasis. These findings expanded the possibility of helicase-like transcription factor as a tumor inhibitor of CRC (21). Although some studies have confirmed the functional changes of a single SNO protein, there is still a lack of relevant studies on the identification of all potential SNO modification sites in human tissues, CRC cell lines, and normal cell lines using proteomics and other high-throughput methods. In addition, research on the SNO modification of tumor proteins has often focused on the activation or inhibition of the tumor cell apoptosis pathway, while there is a lack of relevant studies on the significance of SNO proteins in the tumor endocrine and metabolic pathways. Therefore, research aimed at identifying all potential SNO modification sites in CRC cells and analyzing the endocrine and metabolism-related functions of SNO proteins is particularly important.

In this study, Web of Science and PubMed databases were used to retrieve the studies using key words such as “S-nitrosylation”, “colorectal cancer” and “proteomic”, which identified only four articles. Three of them explored the functional changes of a single protein after SNO modification using CRC cell lines, without using large-scale proteomic analysis. Thus, we only found one relevant study. Chen and co-workers performed site-specific quantification analysis of SNO in tumor and normal tissues from patients with CRC and identified 174 nitrification sites in 94 proteins (13). Therefore, we concluded that research focusing on the differences of SNO proteins in human tissues and CRC cell lines using proteomic analysis is very rare. Furthermore, in almost all the studies concerning SNO, S-nitrosoglutathione (GSNO) and other similar drugs were used as agents to alter the status of SNO, which were added directly into cell culture, often inevitably resulting in additional complicated biological responses. In this study, GSNO was used directly to treat the protein extracts, which would not lead to changes in biological responses. In addition, in previous study, most of the mass spectrometry (MS) results focused on endogenous SNO protein, whereas the proteomic analysis results of this study not only contained the endogenous SNO sites in CRC, but also the potential SNO sites in CRC. According to the results of Gene Ontology (GO) analysis, several SNO proteins, such as glutamate dehydrogenase 1 (GLUD1), pyruvate kinase PKM (PKM), and prostaglandin E synthase 3 (PTGES3), might be involved in the metabolic pathways that are important in tumor endocrine biological processes. We believe that both the endogenous and potential SNO sites identified in this study might have significance in the proliferation and apoptosis of CRC cells, providing the basis for the subsequent study of the stability and functional changes of SNO proteins.

## 2 Materials and methods

### 2.1 Reagents

RIPA lysis buffer and 4 ml, 10 kDa Amicon filters were purchased from Millipore (Billerica, MA, USA). Other reagents were purchased from Sigma-Aldrich (St. Louis, MO, USA), including the protease cocktail inhibitor, 2-[4-(2-hydroxyethyl)-piperazin-1-yl] ethanesulfonic acid (HEPES), ethylene diamine tetraacetic acid (EDTA), neocuproine, sodium dodecyl sulfate (SDS), dimethyl sulfoxide (DMSO), GSNO, methyl methylsulfinylmethyl sulfide (MMTS), N, N-dimethylformamide (DMF), acetone, ascorbic acid (AA), TritonX-100, Tris (2-carboxyethyl) phosphine (TCEP), iodoacetamide, acetonitrile (ACN), and trifluoroacetic acid (TFA) (22). N-[6-(biotinamido)hexyl]-3’-(2’-pyridyldithio)propionamide (Biotin-HPDP) and Dynabeads™ Biotin Binder were purchased from Thermo Fisher Scientific (Waltham, MA, USA).

### 2.2 Cell cultures

Two cell lines, including a normal colonic mucosa cell line (NCM 460) and a CRC cell line (SW 480), were bought from the American Type Culture Collection (Manassas, VA, USA) (23). Cell lines were cultured in Dulbecco’s modified Eagle’s medium (DMEM) (Procell, Austin, TX, USA; catalog number 150210) mixed with both 10% fetal bovine serum (Procell, catalog number 164210-50) and 1% 10 kU/ml penicillin/10 mg/ml streptomycin (Procell, catalog number PB 180120) at 37°C in a humid environment with 5% CO_2_ (24).

### 2.3 Clinical samples

The clinical samples of cancer and para-cancerous tissues were obtained from one patient with colon cancer. The tissue samples were taken from patient at the Colorectal and Anal Surgery Department of the Second Hospital of Jilin University and reviewed by two pathologists for their gastrointestinal pathology. The patient provided informed written consent according to the requirements and approval of the Hospital Research Ethics Committee. In our laboratory, the clinical samples were freshly isolated and stored at -80°C.

### 2.4 Protein extraction

Human tissues were homogenized in RIPA lysis buffer (Millipore, catalog number 20-188) with a 0.2% mixture of protease cocktail inhibitor (Sigma-Aldrich, catalog number PIC 0005) on ice (22). The homogenates were centrifuged at 4°C and 12000 × g for 30 min, and the supernatants were collected. Proteins extracted from SW 480 and NCM 460 cells were prepared by using RIPA lysis buffer on ice for 30 min. The suspensions were centrifuged in 4°C and 12000 × g for 30 min. The protein concentration in the supernatant was determined using the bicinchoninic acid (BCA) method, and the protein samples were stored at -80°C (25).

### 2.5 GSNO pretreatment of protein samples

Protein samples (0.5 mg) were diluted with 1 ml of SNO reaction buffer (250 mM HEPES, 1 mM EDTA, 0.1 mM neocuproine, pH 7.7) for each group. To induce SNO proteins, three groups of protein samples were prepared: The control (GSNO-/AA-) group (added with 10 μl of DMSO), the GSNO-/AA+ group (added with 10 μl of DMSO), and the GSNO+/AA+ group (added with 10 μl of GSNO stock solution (10 mM GSNO in DMSO) to obtain final concentration of 0 μM, 0 μM and 100 μM GSNO in the three groups. The protein samples were incubated at 37°C, and shaken well at 800 rpm for 30 min. After incubation, the protein samples were cooled to 4°C to stop the reaction and minimize the changes in mercaptan modification. The excess GSNO was removed by transferring the sample to 4 ml Amicon filters (Millipore, UFC 8010) and conducting buffer exchanges with 4 ml of cold water at 4°C three times. The protein samples were centrifuged at 4000 × g for 15 min after each exchange. The final sample volume was about 50 μl (26).

### 2.6 Biotin switch technology

The three groups of protein samples were separately diluted to 1.8 ml using HEN buffer (100 mM HEPES, 1 mM EDTA, 0.1 mM neocuproine, pH 8.0) in 15 ml conical tubes. A total of 0.2 ml 25% SDS and 20 μl of 10% MMTS (v/v in DMF) were added to the tubes. The protein samples were incubated in the dark at 50°C for 20 min. Then, a total of 6 ml cold acetone was added to each sample. The protein samples were precipitated at -20°C for 20 min and centrifuged 2000 × g for 5 min. The clear supernatants were discarded after centrifugation and the protein samples were washed using 70% acetone (4 × 5 ml) gently. The protein samples were suspended in 0.24 ml HENS buffer (HEN buffer with 1% SDS (w/v)), and then transferred to 30 μl Biotin-HPDP (Thermo Fisher Scientific, catalog number 21341) (2.5 mg/ml Biotin-HPDP in DMSO) in fresh 1.7 ml microcentrifuge tubes. In order to test the effect of different concentrations of AA on reducing SNO proteins, we carried out the concentration gradient experiment of AA (200 mM, 400mM, 1 M). In the subsequent experiments, a total of 30μl of AA (1 M AA in HEN buffer) were added to the GSNO-/AA+ group and the GSNO+/AA+ group to start the labeling reaction. The equivalent concentration of NaCl was added to the control (GSNO-/AA-) group. Then, a total of 0.9 ml of cold acetone was added to each sample. The protein samples were precipitated at -20°C for 20 min and centrifuged at 5000 × g for 5 min. The clear supernatants were discarded after centrifugation and the protein samples were washed by 70% acetone (4 × 1 ml) gently (27).

### 2.7 Magnetic beads enrichment

Magnetic beads (Dynabeads™ Biotin Binder) were pre-cleaned according to the manufacturer’s instructions. The protein samples from section 2.6 were completely resuspended in 0.25ml HENS/10 buffer (HEN/10 buffer (HEN buffer diluted 10-fold with dH2O) with 1% SDS (w/v)). Thereafter, a total of 0.75 ml neutralization buffer (25 mM HEPES, 100 mM NaCl, 1mM EDTA, 0.5% TritonX-100, pH 7.5) was added to the protein samples. A small portion of each sample (10 μl) was removed for protein “input” analysis. The surplus materials were transferred to 1.7 ml microcentrifuge tubes containing 50 μl of pre-cleaned magnetic beads. The volume of magnetic beads was the same in all samples. The protein samples were rotated gently at 4°C for 12-18 hours (27). The magnetic beads were collected using a magnetic rack for 5 min, the supernatant was removed, and then the magnetic beads were washed using washing buffer (25 mM HEPES, 600 mM NaCl, 1mM EDTA, 0.5% TritonX-100, pH 7.5) (4 × 1 ml). After final cleaning, the supernatants were discarded. Then, a total of 30 μl of 2 × Laemmli loading buffer containing non reducibility were added to the magnetic beads, followed by 6 μl of 50 mM TCEP, and the magnetic beads were incubated at 37°C for 30 min. Then, a total of 6 μl of 100 mM iodoacetamide (avoid light) were added to the magnetic beads, which were incubated at 37°C for 30 min. Finally, the magnetic beads were heated to 95°C for 5 min. After centrifugation, the same amount of the supernatant from each sample was separated by electrophoresis on 10% SDS-polyacrylamide gels. The gels were used subjected to Coomassie blue staining and Western blot. After the Coomassie blue staining, the selected protein bands were excised, destained, and digested using trypsin (37°C, overnight). Peptides were extracted using ACN/0.1% TFA (v/v), dried under a vacuum, and redissolved before identification by MS (28).

### 2.8 MS analysis

**Figure 1:**
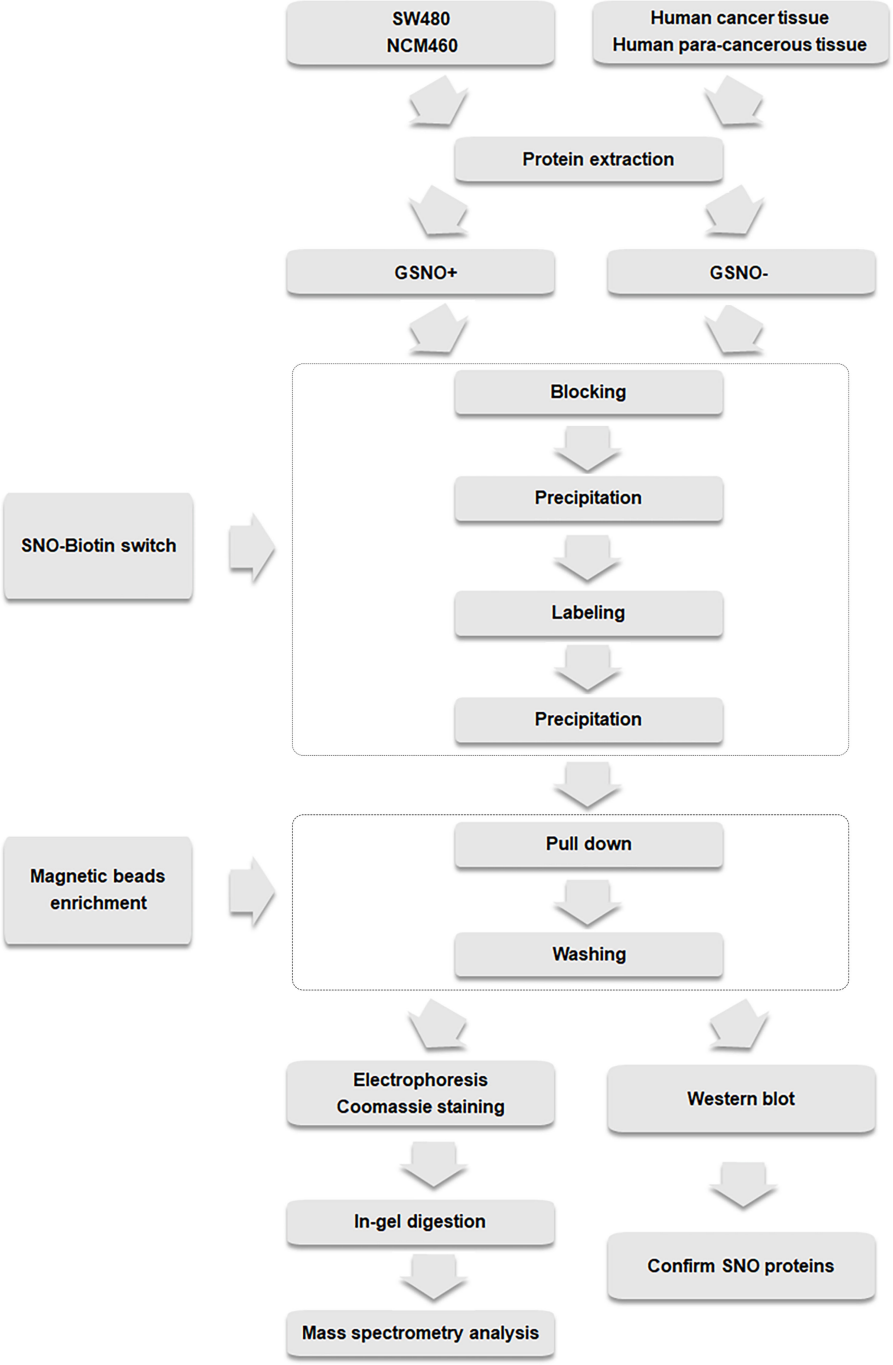
Experimental flow chart of biotin switch and magnetic bead enrichment of potential and endogenous SNO proteins.

An Orbitrap Exploris 480 mass spectrometer (Thermo Fisher Scientific) was used to analyze the separated peptides in a positive ion mode. MS 1 was acquired at a resolution of 60,000 from m/z 350 to 1500. The peptide precursor ions with minimum intensity of 8000 and charge state of 2-6 were selected for MS/MS analysis at a resolution of 15,000 under data-dependent acquisition mode. Higher-energy collisional dissociation (HCD) with normalized collision energy (NCE) of 30, along with an isolation width of 1.6 m/z, was applied. The dynamic exclusion was set as 45 s. The peptide mass spectra and the fragment spectra were collected. Proteome Discoverer 3.0 (Thermo Fisher Scientific) was used to extract the MS/MS spectra from the original data file. The collected data files were searched against the Uniprot database through Proteome Discoverer 3.0. The following search criteria were applied: The proteins were digested by trypsin; two missed cleavages were allowed; carbamidomethyl (C) was set as a fixed modification, whereas oxidation (M) was considered as a variable modification; initial mass deviation of precursor mass tolerance and fragment mass tolerance were allowed up to 10 ppm and 0.02 Da, respectively; and the false discovery rate was 1% (29, 30). The original data was uploaded to the ProteomeXchange Consortium (http://proteomecentral.proteomexchange.org) (31) with data identification number PXD 039376. The general flowchart of the experiment was shown as follows (**Figure 1**). For biological processes, all identified proteins were analyzed using the GO analysis.

## 3 Results

### 3.1 Enrichment of SNO proteins using biotin switch technology

**Figure 2:**
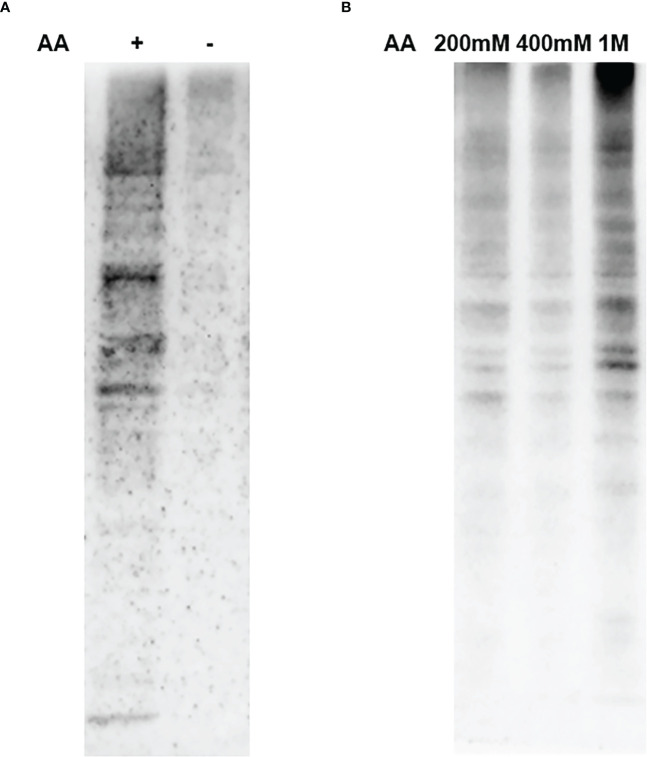
Results of AA reducing SNO proteins. **(A)** The effect of AA on reducing SNO proteins. **(B)** The reduction effect of different AA concentrations (200 mM, 400 mM, 1 M) on SNO proteins.

To clarify the role of AA in the biotin switch method, SW 480 cells were used to conduct a Western blot experiment to enrich endogenous SNO proteins. The results confirmed that AA obviously reduced the extent of SNO reducing the -SNO bonds of proteins to -SH bonds (**Figure 2A**). AA was necessary in the biotin switch technology to enrich the SNO proteins. Furthermore, in order to find the most effective experimental concentration of AA, we conducted a concentration gradient experiment. With the increase in AA concentration (200 mM, 400 mM, 1 M), the amount of reduced SNO proteins increased gradually (**Figure 2B**).

### 3.2 Differences of SNO sites identification between CSNO+ group and GSNO- group in human tissues and cell lines

**Figure 3:**
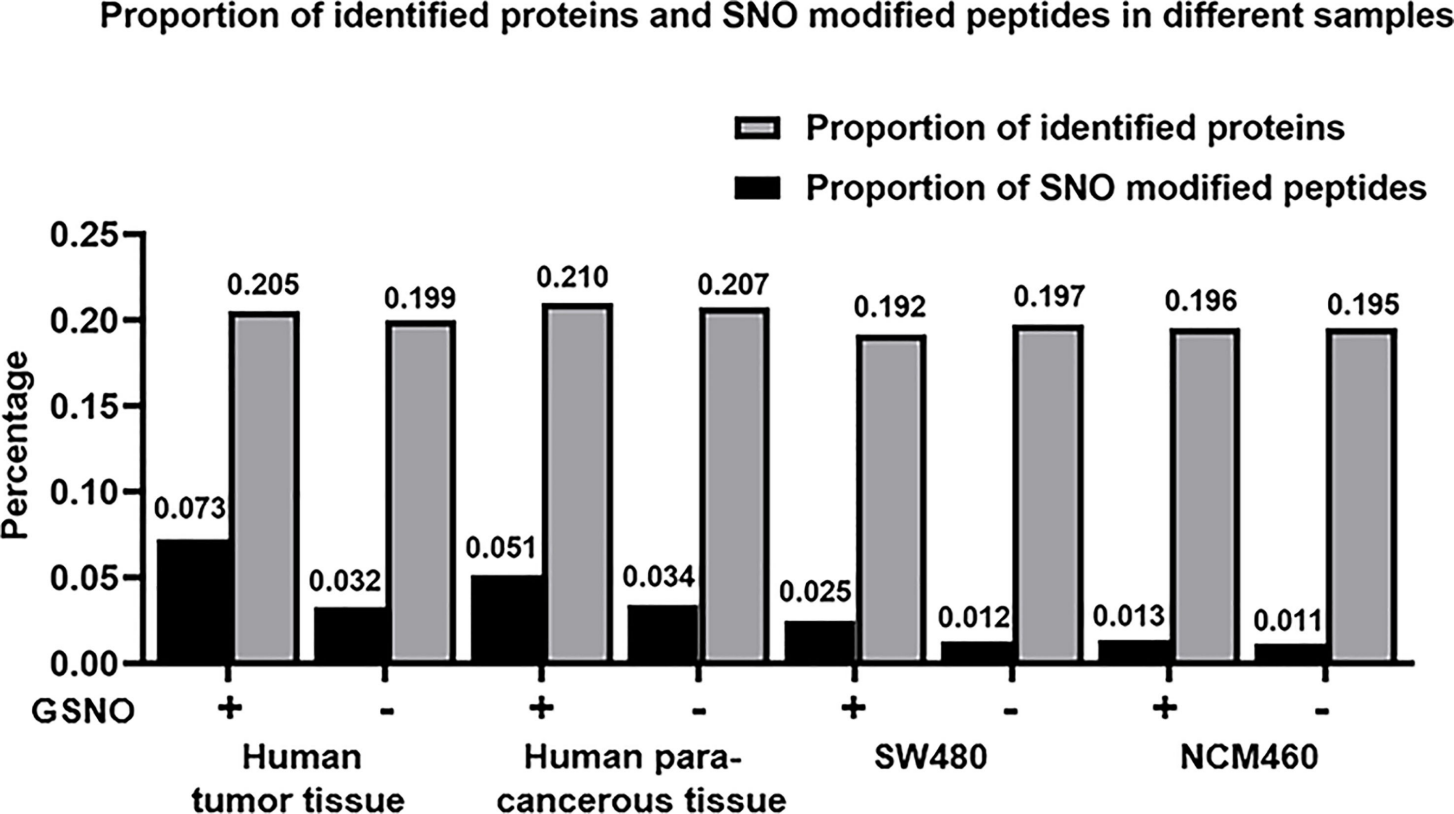
The proportion of SNO peptides and identified proteins in different protein samples.

Prior to MS analysis, the human tissue and cell line protein samples were processed by biotin switch technology. A total of 6581 proteins were detected in the GSNO+ sample of human cancer tissue, and 4067 proteins were detected in the GSNO- sample of human cancer tissue. For the human para-cancerous tissue, a total of 2567 proteins were detected in the GSNO+ group and 1727 proteins were detected in the GSNO- group. In addition, a total of 8357 proteins were detected in the GSNO+ group of SW 480 cells, and 9515 proteins were detected in the GSNO- group of SW 480 cells. For NCM 460 cells, a total of 6651 proteins were detected in the GSNO+ group and 9143 proteins were detected in the GSNO- group. However, the number of identified proteins which was shown in MS results, was much smaller than the number of the total detected proteins, and the number of SNO modified proteins was much smaller than the number of identified proteins which was shown in MS results. The proportions of identified proteins which was shown in MS results, and SNO modified peptides were shown in **Figure 3**. The above results indicated that the endogenous SNO modified peptides represented about 1-2% of the total peptides for SW 480 and NCM 460 cells, whereas the endogenous SNO modified peptides in human tissue represented over 3% of the total peptides. Furthermore, the results indicated that there was a significant proportion of potential SNO sites detected in this study. On the one hand, the percentage of identified proteins was similar in the GSNO+ and GSNO- groups of all sample types (19-20%). On the other hand, the number of SNO peptides was higher in the GSNO+ groups than that in the GSNO- groups. In addition, the percentage of both total proteins and SNO peptides were higher in human tissues compared with those in cell lines. The potential SNO sites might have important implications in the genesis and progression of CRC.

Although the SNO proteins accounted for a low proportion of all tumor proteins, many SNO proteins were identified in this study. Among them, a total of 233 proteins were identified in human cancer tissue, which had potential or endogenous SNO modification sites. More accurately, a total of 428 peptide segments and 227 proteins were identified in the GSNO+ group of human cancer tissue, which had potential SNO sites. In contrast, a total of 91 peptide segments and 59 proteins were identified in the GSNO- group of human cancer tissue, which had endogenous SNO sites (**Figure S1A**). For the human para-cancerous tissue, a total of 46 proteins were identified with potential or endogenous SNO modification sites. In the GSNO+ group of human para-cancerous tissues, a total of 78 peptide segments and 41 proteins were identified with potential SNO sites. In the GSNO- group of human para-cancerous tissue, a total of 32 peptide segments and 22 proteins were identified with endogenous SNO sites (**Figure S1B**). However, fewer SNO proteins were identified in cell lines compared with that in human tissue. In SW 480 cells, a total of 115 proteins were identified with potential or endogenous SNO sites. Among them, a total of 129 peptide segments and 99 proteins containing potential SNO sites were identified in the GSNO+ group of SW 480 cells. Whereas, in the GSNO- group of SW 480 cells, a total of 70 peptide segments and 57 proteins were identified with endogenous SNO sites (**Figure S1C**). For NCM 460 cells, a total of 62 proteins were identified that had potential or endogenous SNO sites. In the GSNO+ group of NCM 460 cells, a total of 46 peptide segments and 39 proteins were identified with potential SNO sites. In contrast, a total of 57 peptide segments and 44 proteins were identified with endogenous SNO sites in the GSNO- group of NCM 460 cells (**Figure S1D**). In the MS results for human tissue and cell lines, some peptides, which were present in the GSNO+ group and the GSNO- group at the same time, might have both endogenous SNO sites and cysteine residues (-SH) that might be modified by nitrosos. In addition, potential SNO peptides were identified only in the GSNO+ group, suggesting that these peptides had cysteine residues (-SH), and had the potential to be modified by SNO under specific conditions. Potential SNO peptides and proteins might play an important role in CRC. SNO peptides displayed in both the GSNO+ group and the GSNO- group suggesting that these peptides had endogenous SNO sites, but might concurrently have other cysteine residues (-SH) that might be modified by nitrosos. Correspondingly, the SNO peptides were only identified in the GSNO- group, suggesting that these peptides had endogenous SNO sites and might not have any cysteine residues (-SH) that might be modified by nitrosos. These results proved the existence of endogenous SNO proteins and the differential abundance of SNO proteins in tumor cells and normal cells. The results provided a possible research basis to study the effect of SNO proteins on the malignant transformation processes of normal cells.

### 3.3 Potential and endogenous SNO peptides and special SNO proteins detected in human tissues and cell lines

**Figure 4:**
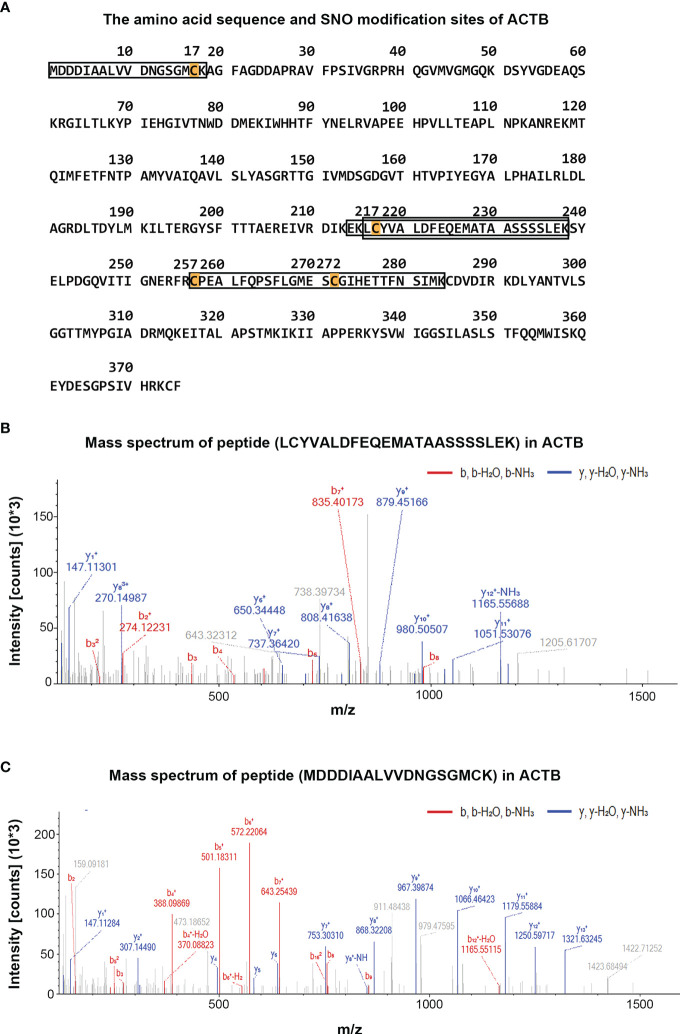
Display diagram of SNO modification sites of ACTB and mass spectra diagram of SNO peptides of ACTB in different samples. **(A)** The detected amino acid sequence and SNO modification sites of ACTB. **(B)** The mass spectra diagram of peptide (LcYVALDFEQEMATAASSSSLEK) of ACTB identified in human cancer tissue. **(C)** The mass spectra diagram of peptide (mDDDIAALVVDNGSGMcK) of ACTB identified in SW 480 cells.

By comparing the MS data of human tissues and cell lines, a total of 19 proteins were identified that appeared in both human cancer tissue and SW 480 cells containing potential or endogenous SNO sites (**Table 1**). Therefore, these 19 proteins had high reliability. Among them, several SNO proteins were detected with completely different SNO peptides and sites in different samples, such as actin, cytoplasmic 1 (ACTB), cofilin-1 (CFL1), GAPDH, malate dehydrogenase, mitochondrial (MDH2), mitochondrial 60 kDa heat shock protein (HSPD1), and peroxiredoxin-4 (PRDX4) (**Table 2**). The SNO proteins were detected in both SW 480 cells and human cancer tissue, suggesting that these proteins might have important implications in the genesis, progression and apoptosis of CRC, such as nucleoside diphosphate kinase (NME1-NME2), ACTB (**Figures 4A–C**), and protein S100A8 (S100A8).

**Table 1:** List of 19 SNO proteins identified in both human cancer tissue and SW 480 cells.

No.	Peptide	Accession	Description	MW [kDa]	calc. pI	Gene Symbol	Human cancer tissue		SW 480	
							GSNO+	GSNO-	GSNO+	GSNO-
1	mDDDIAALVVDNGSGMcK	P60709	Actin, cytoplasmic 1	41.7	5.48	ACTB	•	•	•	•
2	ELPPDQAEYcIAR	P12814	Alpha-actinin-1	103	5.41	ACTN1	•	•	•	•
3	ELPPDQAEYcIAR	O43707	Alpha-actinin-4	104.8	5.44	ACTN4	•	•	•	•
4	mSTVHEILcK	P07355	Annexin A2	38.6	7.75	ANXA2	•	•	•	•
5	HELQANcYEEVKDR	P23528	Cofilin-1	18.5	8.09	CFL1	•	•	•	•
6	IISNAScTTNcLAPLAK	P04406	Glyceraldehyde-3-phosphate dehydrogenase	36	8.46	GAPDH	•	•	•	•
7	AScLYGQLPK	P09211	Glutathione S-transferase P	23.3	5.64	GSTP1	•	•	•	•
8	AAVEEGIVLGGGcALLR	P10809	60 kDa heat shock protein, mitochondrial	61	5.87	HSPD1	•	•	•	•
9	VIGSGcNLDSAR	P00338	L-lactate dehydrogenase A chain	36.7	8.27	LDHA	•	•	•	•
10	VIGSGcNLDSAR	P07195	L-lactate dehydrogenase B chain	36.6	6.05	LDHB	•	•	•	•
11	DSNNLcLHFNPR	P09382	Galectin-1	14.7	5.5	LGALS1	•	•	•	•
12	TIIPLISQcTPK	P40926	Malate dehydrogenase, mitochondrial	35.5	8.68	MDH2	•	•	•	•
13	GDFcIQVGR	Q32Q12	Nucleoside diphosphate kinase	32.6	8.48	NME1-NME2	•	•	•	•
14	IIPGFMcQGGDFTR	P62937	Peptidyl-prolyl cis-trans isomerase A	18	7.81	PPIA	•	•	•	•
15	TREEEcHFYAGGQVYPGEASR	Q13162	Peroxiredoxin-4	30.5	6.29	PRDX4	•	•	•	•
16	LLETEcPQYIR	P05109	Protein S100-A8	10.8	7.03	S100A8	•	•	•	•
17	EAGDVcYADVYR	J3KTL2	Serine/arginine-rich-splicing factor 1	28.3	10.08	SRSF1	•	•	•	•
18	VPADTEVVcAPPTAYIDFAR	P60174	Triosephosphate isomerase	30.8	5.92	TPI1	•	•	•	•
19	ScSGVEFSTSGSSNTDTGK	P45880-1	Isoform 1 of Voltage-dependent anion-selective channel protein 2	33.4	7.59	VDAC2	•	•	•	•

c, the site with modification of carbamidomethyl. m, the site with modification of methylthio.

**Table 2:** List of different SNO sites of the same protein in the above 19 proteins.

Accession	Description	Gene Symbol	Peptide	Site	Human cancer tissue		Human para-cancerous tissue		SW 480		NCM 460	
					GSNO+	GSNO-	GSNO+	GSNO-	GSNO+	GSNO-	GSNO+	GSNO-
P60709	Actin, cytoplasmic 1	ACTB	mDDDIAALVVDNGSGMcK	C17	•	•			•	•	•	•
			EKLcYVALDFEQEMATAASSSSLEK	C217	•							
			LcYVALDFEQEMATAASSSSLEK	C217	•	•						
			cPEALFQPSFLGMEScGIHETTFNSIMK	C257, C272	•							
P12814	Alpha-actinin-1	ACTN1	cQLEINFNTLQTK	C332	•							
			DGLGFcALIHR	C180	•							
			EGLLLWcQR	C154	•							
			IcDQWDNLGALTQK	C480	•							
			ELPPDQAEYcIAR	C860	•	•	•		•	•		
O43707	Alpha-actinin-4	ACTN4	cQLEINFNTLQTK	C351	•							
			EGLLLWcQR	C173	•							
			IcDQWDALGSLTHSR	C499	•							
			ELPPDQAEYcIAR	C879	•	•	•		•	•		
P07355	Annexin A2	ANXA2	mSTVHEILcK	C9	•	•			•	•	•	•
			GLGTDEDSLIEIIcSR	C13	•							•
P23528	Cofilin-1	CFL1	AVLFcLSEDKK	C39	•							
			HELQANcYEEVKDR	C139	•	•			•	•	•	•
			KAVLFcLSEDKK	C39	•							
P04406	Glyceraldehyde-3-phosphate dehydrogenase	GAPDH	IISNAScTTNcLAPLAK	C152, C156	•	•			•	•	•	•
			VPTANVSVVDLTcR	C247	•	•	•		•	•	•	•
			VPTANVSVVDLTcRLEKPAK	C247					•			
P10809	60 kDa heat shock protein, mitochondrial	HSPD1	cEFQDAYVLLSEKK	C237	•				•	•	•	•
			AAVEEGIVLGGGcALLR	C442	•	•			•	•	•	•
P09382	Galectin-1	LGALS1	DSNNLcLHFNPR	C43	•	•	•		•	•		
			FNAHGDANTIVcNSK	C61	•	•						
P40926	Malate dehydrogenase, mitochondrial	MDH2	EGVVEcSFVK	C275	•				•			
			GcDVVVIPAGVPR	C93	•							
			GYLGPEQLPDcLK	C89	•	•			•			
			SQETEcTYFSTPLLLGKK	C285	•							
			TIIPLISQcTPK	C212	•	•			•	•		
			SQETEcTYFSTPLLLGK	C285	•	•			•			
P62937	Peptidyl-prolyl cis-trans isomerase A	PPIA	HTGPGILSmANAGPNTNGSQFFIcTAK	C115	•							
			HTGPGILSMANAGPNTNGSQFFIcTAK	C115	•				•	•		
			KITIADcGQLE	C161	•				•	•	•	•
			IIPGFmcQGGDFTR	C62	•	•	•				•	•
			IIPGFMcQGGDFTR	C62	•	•	•		•	•		
Q13162	Peroxiredoxin-4	PRDX4	TREEEcHFYAGGQVYPGEASR	C51	•	•			•	•		•
			HGEVcPAGWKPGSETIIPDPAGK	C245					•	•		
			SINTEVVAcSVDSQFTHLAWINTPR	C148					•	•		
P05109	Protein S100-A8	S100A8	KLLETEcPQYIR	C42	•				•			
			LLETEcPQYIRK	C42	•	•	•	•	•	•	•	•
P60174	Triosephosphate isomerase	TPI1	DcGATWVVLGHSER	C124	•		•		•		•	
			DcGATWVVLGHSERR	C124	•							
			IAVAAQNcYK	C104	•				•		•	
			VAHALAEGLGVIAcIGEKLDER	C164	•							
			VPADTEVVcAPPTAYIDFAR	C79	•	•			•	•	•	•
			IIYGGSVTGATcK	C255			•		•		•	
			VAHALAEGLGVIAcIGEK	C164					•			
P45880-1	Isoform 1 of Voltage-dependent anion-selective channel protein 2	VDAC2	ScSGVEFSTSGSSNTDTGK	C62	•	•			•	•	•	•
			WcEYGLTFTEK	C91	•				•			
			WNTDNTLGTEIAIEDQIcQGLK	C118	•							
			YKWcEYGLTFTEK	C92	•							
P09211	Glutathione S-transferase P	GSTP1	AScLYGQLPK	C480	•	•			•	•	•	•
P00338	L-lactate dehydrogenase A chain	LDHA	VIGSGcNLDSAR	C163	•	•			•	•		
P07195	L-lactate dehydrogenase B chain	LDHB	VIGSGcNLDSAR	C164	•	•			•	•		
Q32Q12	Nucleoside diphosphate kinase	NME1-NME2	GDFcIQVGR	C134, C249	•	•			•	•	•	•
J3KTL2	Serine/arginine-rich-splicing factor 1	SRSF1	EAGDVcYADVYR	C148	•	•			•	•	•	•

c, The site with modification of carbamidomethyl. m, The site with modification of methylthio.

### 3.4 Biological processes associated with endogenous SNO proteins according to GO analysis

**Figure 5:**
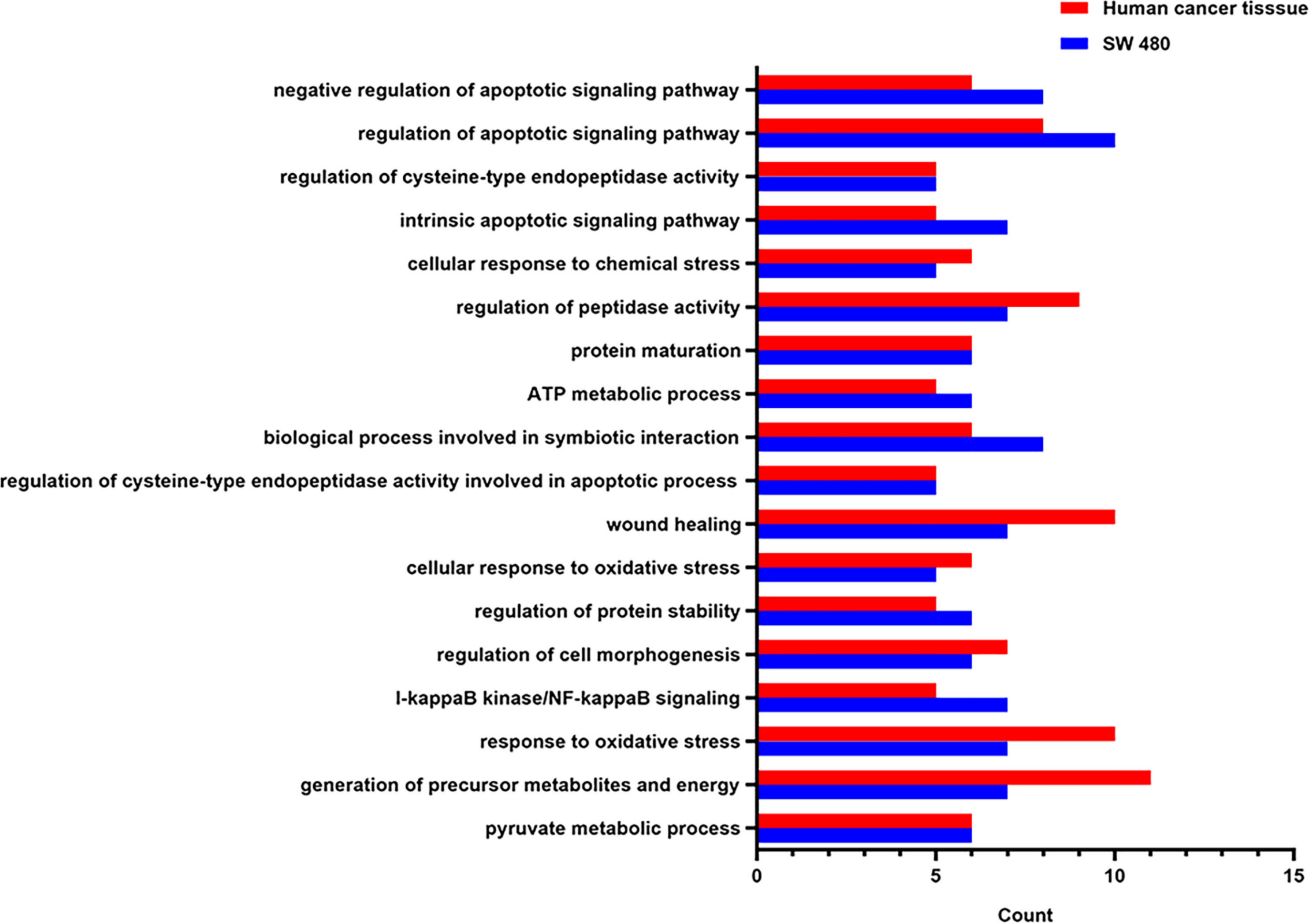
The top 12 most significant biological processes involved by SNO proteins.

**Figure 6:**
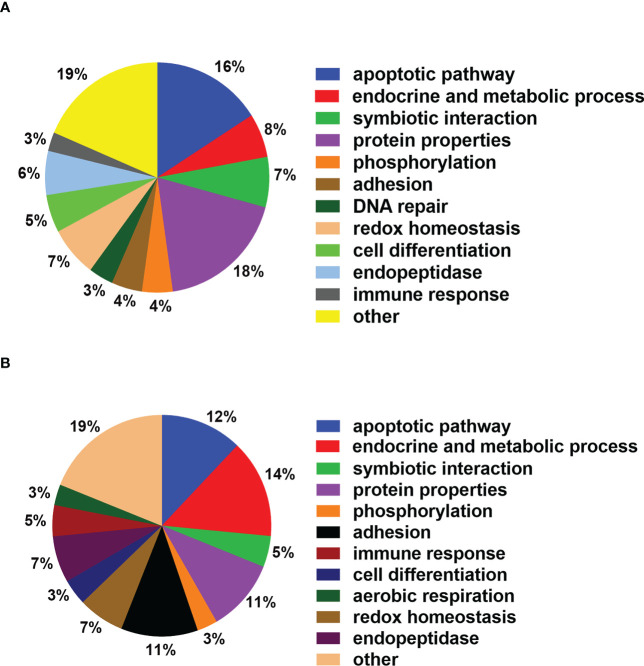
Main biological functions of SNO proteins. **(A)** Proportion of SNO protein detected in SW 480 participating in main biological processes. **(B)** Proportion of SNO protein detected in human cancer tissue participating in main biological processes.

To clarify the potential functional roles of the endogenous SNO proteins in CRC, annotations of subcellular function were performed using GO analysis. A total of 134 biological processes were associated with endogenous SNO proteins in human cancer tissue and 131 biological processes were associated with endogenous SNO proteins in SW 480 cells (**Table S1**). Among them, endogenous SNO proteins were closely related to 59 biological processes in both human cancer tissue and SW 480 cells. According to the number of proteins involved in biological processes exceeding 5, only the top 12 most significant GO terms were used to determine the general function of the endogenous SNO proteins in this study (**Figure 5**). The functional analysis of SNO proteins in SW 480 cells and human cancer tissue showed that the top three biological processes were cell apoptosis, tumor endocrine and metabolic pathways, and protein properties (stability and maturation of proteins) (**Figures 6A, B**).

According to the GO results, catalase (CAT), peroxiredoxin-1 (PRDX1), and PRDX4 were related to hydrogen peroxide catabolic process and reactive oxygen species metabolic process, which led to tumor cell apoptosis. Fibrinogen beta chain (FGB), filamin-A (FLNA), and fibrinogen gamma chain (FGG) were associated with homotypic cell-cell adhesion and regulation of substrate adhesion-dependent cell spreading, which were closely related to the invasion and metastasis of tumor cells. Alcohol dehydrogenase 1A (ADH1A), CAT, GAPDH, and MDH2 were related to the generation of precursor metabolites and energy, which played an important role in the proliferation of tumor cells. FLNA, GAPDH, glutathione S-transferase P1 (GSTP1), and galectin-1 (LGALS1) were related to the I-kappaB kinase/NF-kappaB signaling pathway, which was closely related to the apoptosis process of tumor cells. Cell division control protein 42 homolog (CDC42), GSTP1, and peptidyl-prolyl cis-trans isomerase A (PPIA) were associated with regulation of the stress-activated mitogen activated protein kinase (MAPK) cascade, which were closely related to the tumor cell proliferation.

Thus, the GO analysis showed that SNO proteins might play important roles in the proliferation, metastasis, and apoptosis of tumor cells and were related to the tumor endocrine biological processes and metabolic pathways. According to the GO analysis, ACTB was associated with the regulation of body fluid levels and the positive regulation of norepinephrine uptake. Alpha-1-antitrypsin (SERPINA1), another SNO protein, was related to the regulation of body fluid levels and the acute inflammatory response. In addition, S100A8 was associated with the response to lipopolysaccharide, leukocyte aggregation, and the acute inflammatory response. The SNO proteins isocitrate dehydrogenase [NADP], mitochondrial (IDH2), MDH2, and succinate dehydrogenase [ubiquinone] flavoprotein subunit, mitochondrial (SDHA) were related to the tricarboxylic acid cycle. GSTP1 was related to the prostaglandin metabolic process and the acute inflammatory response, and PPIA was associated with the regulation of body fluid levels. The SNO proteins GAPDH, L-lactate dehydrogenase A chain (LDHA), PKM, and triosephosphate isomerase (TPI1) were closely related to the carbohydrate catabolic process. In addition, SNO LDHA and L-lactate dehydrogenase B chain (LDHB) were closely related to the lactate metabolic process. Lastly, GO annotation suggested that SNO PTGES3 played an important role in the prostaglandin metabolic process.

## 4 Discussion

As an important part of epigenetics, post-translational modification has important implications in the genesis and progression of tumors. Currently, research is focused on phosphorylation and ubiquitination. Although the proportion of SNO modifications among the overall post-translational modifications is smaller than that of phosphorylation and ubiquitination (16), SNO proteins still have important biological functions and significance in, for example, CRC (32), breast cancer (33), and gastric cancer (34).

Through comparative analysis using the endogenous human S-nitroso protease map, the first 17 SNO proteins with high expression were provided by Chen, et al. (13). In the present study, we found that PKM and ANXA4 were detected with SNO in human cancer tissue. Moreover, SNO of HSPD1 and PRDX4 were detected in SW 480 cells and human cancer tissue. Therefore, it is possible that SNO modification of the above four proteins has important implications in the genesis and progression of CRC. HSPD1 is considered to be capable of catalyzing the SNO modification under the regulation of iNOS. Overexpression of iNOS leads to a high concentration of NO, thus inducing the HSPD1 to participate in the development of CRC (13). The nitrosylation site on Cys237 marks it as a key residue in the interaction between S-nitrosylated HSPD1 and iNOS, which regulates the mitochondrial biogenesis and intra-mitochondrial transport induced by inflammation (13). ANXA4 might be a potential tumor biomarker of CRC. High expression of ANXA4 is closely related to the low survival of patients with CRC (13). However, the potential correlation between SNO of ANXA4 and the cancer mechanism has not been reported. In addition, in this study, the protein samples were enriched by magnetic beads and then subjected to gel hydrolysis and enzymatic hydrolysis, while Chen first hydrolyzed the SNO proteins and then enriched them using streptavidin agarose beads. In addition, Chen et al. used the Q-TOF Premier mass spectrometer, while we used Orbitrap Explorer 480 mass spectrometer. More importantly, the difference between individuals and cell lines is an unavoidable problem in biological research. In this study, SW 480 cells were used for MS analysis in this study, while in the study of Chen et al., SNO proteins of CC-M1 cell line were detected using MS analysis. These methodological differences might explain the differences in the SNO peptide detection results between the present study and that of Chen et al.

In this study, several proteins identified in different samples had different SNO peptides and sites, such as ACTB, PRDX4, GAPDH, S100A8, and annexin A2 (ANXA2). Herein, the SNO modification process was carried out in the protein extracts. Compared with the complex biological process carried out in CRC cells, our experimental conditions of SNO modification are more stable. However, there are still folding and helical spatial conformations in the proteins, and these complex spatial structures might represent a huge obstacle to the SNO modification of proteins. In addition, the enrichment and purification of SNO proteins depended on the dual effects of GSNO and AA. The randomness of the combination of GSNO and cysteine residues, and AA and SNO proteins, is also an important reason for the detection of different SNO peptides and sites of the same protein, and thus might not represent a genuine biological difference, which is a limitation of our study. Proteins are usually composed of multiple peptide segments, and the degree of peptide fragmentation differs because of temperature, humidity, reaction time, and pH value in the process of enzymatic hydrolysis of proteins. Therefore, this can also lead to differences in the results of the MS analysis of the same protein. ACTB, an important component of the cytoskeleton that plays a crucial role in cell growth and migration, is significantly overexpressed in CRC cell lines with strong invasion ability (35). The expression change of ACTB is directional and correlates positively with the clinical activity of CRC progression (36). Ahlquist et al.’s predicted CRC by detecting methylation of the ACTB, and the results confirmed that the expression of ACTB has good sensitivity and specificity in predicting CRC (37). ACTB might also regulate tumor metastasis and invasion through the NF-κB and Wnt/β-catenin pathways (35). Further studies are needed to prove the significance of the SNO modification of ACTB in CRC. In advanced CRC, PRDX4 plays an important role in the growth, invasion, and migration of tumor cells, indicating that PRDX4 promotes cell growth, cell cycle distribution, and human cancer progression (38). PRDX4 participates in the progression of CRC by regulating the redox balance, oxidative protein folding and hydrogen peroxide signaling. In addition, the high expression of PRDX4 in CRC tissues is usually associated with an increased risk of liver metastasis (38). In this study, SNO modification of PRDX4 was detected in several samples. However, the role of SNO of PRDX4 in the regulation of CRC has not been determined per now.

The S100 protein family has been identified as a potential marker of CRC, melanoma, bladder cancer and other cancers. S100A8 is a potential marker of gastric cancer and CRC (39). The abnormal expression of S100A8 and unresponsive inflammation can lead to the occurrence of cancer, however, the mechanism of inflammation developing into CRC is still not completely clear (40). In this study, S100A8 was identified in all samples, so we had grounds to believe that S100A8 played an important role in CRC. Research by Ichikawa et al. showed that S100A8/A9 interacted with carboxylated glycans on colon tumor cells and promoted the MAPK and NF-κB signaling pathways (41). Lim et al. believed that monocytes and macrophages in the metastatic liver microenvironment could induce the expression of S100A8 in cancer cells (42). Although S100A8 is not responsible for stimulating cancer cell proliferation, it plays an important role in tumor cell invasion. Lim et al. believed that S100A8 had therapeutic potential to interfere with tumor metastasis (42). Zhang et al. found that S100A8 was induced by inflammation and promoted the development of CRC by activating differentiation inhibitor 3. Zhang et al. also believed that S100A8 could lead to the proliferation and invasion of CRC by recruiting macrophages (40). Finally, Zhang et al. revealed the potential mechanism by which inflammation-induced S100A8 could promote the development of CRC by activating the protein kinase B 1 (Akt1)-Smad family member 5 (Smad5)-inhibitor of DNA binding 3 (Id3) axis (40).

The endocrine and metabolic pathways play an important role in many biological processes in tumor cells and they are closely related to inflammation, energy, and oxidative stress. Some SNO modified proteins detected in this study, such as PKM, GAPDH, LDHA, GLUD1, PPIA, and PTGES3, have been proven to be closely related to the endocrine and metabolic pathways. The metabolic phenotype of some cancer cells is characterized by increased glycolysis and lactic acid production, rather than oxidative phosphorylation of mitochondria (29). Pyruvate kinase is one of the rate-limiting enzymes in glycolysis. PKM is one of the subtypes of pyruvate kinase, and its isozyme selection is related to the metabolic phenotype of cancer cells (43). The re-expression of PKM2 exists in almost all cancers and PKM2 is responsible for maintaining glycolysis-dominated energy metabolism. Therefore, PKM2 is a key factor in increased glycolysis and can promote the selective proliferation of tumors *in vivo* (29). Siragusa et al. suggested that the interaction between active endothelial nitric oxide synthase and S-nitrite PKM2 could reduce the activity of PKM2. Siragusa et al. also found that preventing the reduction of SNO of PKM2 could enhance the antioxidant response of endothelial cells and that SNO of PKM2 could delay the development of cardiovascular disease (44). However, although proteomic methods have identified the SNO of many proteins such as PKM2, so far there has been no research to demonstrate the actual impact of SNO of PKM in CRC in detail.

PKM, as one of the necessary rate-limiting enzymes for glycolysis, plays an important role in the tumor endocrine and metabolic pathways, while GAPDH, as an important link in the glycolysis pathway, is also important in mitochondrial function. The SNO of GAPDH was detected in both human cancer tissue and SW 480 cells in comparison with the list of SNO proteins listed by Kone (17) and our MS results. These results suggested that SNO modified GAPDH might have important implications in the genesis and progression of CRC. Traditionally, studies of GAPDH have mainly focused on its glycolytic function. With the discovery of GAPDH in cytoplasm, mitochondria, nucleus, and other cell compartments, it has gradually been accepted as a multifunctional protein. For example, GAPDH might act as an intracellular sensor for oxidative stress at the early stage of apoptosis. Oxidative stress can often affect the expression and function of nuclear GAPDH, leading to apoptosis (45). The increase in nuclear GAPDH seems to be an upstream event of its apoptotic signaling. NO stress-mediated GAPDH modification seems to target the nucleus (45). An NO donor can stimulate the accumulation of nuclear GAPDH, and the SNO modification can lead to the nuclear translocation of GAPDH, such that it can affect cell apoptosis (45). Li et al. found that microcystins-LR induced NO production in SW 480 cells and promoted the SNO modification of GAPDH. Li et al. also found that the mechanism by which microcystins-LR induced SW 480 apoptosis acted through the SNO-GAPDH-Siah1 cascade signal pathway (19). Further studies of the specific mechanism of the effect of SNO modification on GAPDH stability and function are required.

Currently, research on the role of PKM and GAPDH in the tumor endocrine and glucose metabolism pathways is relatively mature, while there has been less research on the significance of the insulin metabolism pathway, prostaglandin metabolism pathway, and other endocrine pathways in the field of CRC, thus the biological significance of tumor endocrine pathways has deserved more attention in the future. For example, PKM and LDHA are related to central carbon metabolism in cancer. PKM and LDHA can affect the production and metabolism of pyruvate and lactic acid, thus affecting the citric acid cycle and leading to changes in the concentration of reactive oxygen species, thus activating the HIF-1 signaling pathway (46). In addition, the SNO modification of GLUD1 was detected in this study, GLUD1 plays an important role in insulin homeostasis. Deficiency of GLUD1 can lead to abnormal insulin secretion by regulating the sensitivity of guanosine triphosphate (GTP) inhibition, which might affect the balanced regulation between insulin, glucose and the insulin-like growth factor (IGF), which is related to the JAK/STAT and Ras signaling pathways (6, 47). SNO proteins not only affect the glucose metabolism pathway by regulating insulin level, but also participate in inflammatory reactions by regulating prostaglandins. For the acute inflammation, PPIA is closely related to the inflammatory response. PPIA promotes CRC by regulating the inflammatory response of inflammatory bowel disease (36). In addition, the level of prostaglandin is often elevated, and prostaglandin E2 is thought to inhibit apoptosis of cancer cells, and increase tumor invasion and angiogenesis through the NF-κB, MAPK/JNK/p38, and VEGF/PI3kinase/Akt/mTOR pathways and epigenetic modification (7, 8). Levels of PTGES3, which converts prostaglandin endoperoxide H2 to prostaglandin E2, were significantly higher in CRC tissues as opposed to normal tissues (48). The SNO of PTGES3 was identified in SW 480 cells but not in NCM 460 cells, suggesting that the endocrine and inflammatory metabolic pathways play an important role in the occurrence and development of CRC.

## 5 Conclusion

We processed human tissues and related cell lines through biotin switch technology and magnetic beads enrichment, ultimately obtaining nearly 100 SNO peptides and proteins through MS analysis. According to the results of MS and GO analysis, we proved that different CRC cells and tissues contain both potential and endogenous SNO modified proteins. In addition, some SNO proteins can participate in the proliferation, metastasis, and apoptosis of CRC by regulating the tumor endocrine and metabolic pathways, such as PKM, GAPDH, LDHA, GLUD1, and PTGES3. Therefore, we believe that the SNO proteins identified in this study will provide a research basis for the academic community to gain a deeper understanding of biological processes such as the tumor endocrine and metabolism and apoptotic signaling pathways.

## Data availability statement

The datasets presented in this study can be found in online repositories. The names of the repository/repositories and accession number(s) can be found in the article/**Supplementary Material**.

## Ethics statement

The studies involving human participants were reviewed and approved by the Research Ethics Committee of Second Affiliated Hospital of Jilin University. The patients/participants provided their written informed consent to participate in this study.

## Author contributions

FL and SW contributed equally to this work and share first authorship. FL wrote the manuscript. FL, SW, YG, and YM performed the study. FL, FJS, and SW processed the data. SW and MW revised the manuscript. All authors contributed to the article and approved the submitted version.

## Glossary

**Table:**

CRC	colorectal cancer
TNM	tumor, lymph node, and metastasis
SNO	S-nitrosylation
NO	nitric oxide
NF-κB	nuclear factor kappa B
GAPDH	glyceraldehyde-3-phosphate dehydrogenase
Siah1	Siah E3 ubiquitin protein ligase 1
ANXA4	annexin A4
iNOS	inducible nitric oxide synthase
GSNO	S-nitrosoglutathione
MS	mass spectrometry
GO	Gene Ontology
GLUD1	glutamate dehydrogenase 1
PKM	pyruvate kinase PKM
PTGES3	prostaglandin E synthase 3
HEPES	2-[4-(2-hydroxyethyl)piperazin-1-yl] ethanesulfonic acid
EDTA	ethylene diamine tetraacetic acid
SDS	sodium dodecyl sulfate
DMSO	dimethyl sulfoxide
MMTS	methyl methylsulfinylmethyl sulfide
DMF	N, N-dimethylformamide
AA	ascorbic acid
TCEP	Tris(2-carboxyethyl) phosphine
ACN	acetonitrile
TFA	trifluoroacetic acid
Biotin-HPDP	N-[6-(biotinamido)hexyl]-3’-( 2’-pyridyldithio)propionamide
DMEM	Dulbecco’s modified Eagle’s medium
BCA	bicinchoninic acid
AA	ascorbic acid
HCD	higher-energy collisional dissociation
NCE	normalized collision energy
ACTB	actin, cytoplasmic 1
CFL1	cofilin-1
MDH2	malate dehydrogenase, mitochondrial
HSPD1	mitochondrial 60 kDa heat shock protein
PRDX4	peroxiredoxin-4
NME1-NME2	nucleoside diphosphate kinase
S100A8	protein S100A8
CAT	catalase
PRDX1	peroxiredoxin-1
FGB	fibrinogen beta chain
FLNA	filamin-A
FGG	fibrinogen gamma chain
ADH1A	alcohol dehydrogenase 1A
GSTP1	glutathione S-transferase P
LGALS1	galectin-1
CDC42	cell division control protein 42 homolog
PPIA	peptidyl-prolyl cis-trans isomerase A
MAPK	mitogen activated protein kinase
SERPINA1	Alpha-1-antitrypsin
IDH2	isocitrate dehydrogenase [NADP], mitochondrial
SDHA	succinate dehydrogenase [ubiquinone] flavoprotein subunit, mitochondrial
LDHA	L-lactate dehydrogenase A chain
TPI1	triosephosphate isomerase
LDHB	L-lactate dehydrogenase B chain
ANXA2	annexin A2
Akt1	protein kinase B 1
Smad5	Smad family member 5
Id3	inhibitor of DNA binding 3
GTP	guanosine triphosphate
IGF	insulin-like growth factor

## References

[1] Boyaval F van Zeijl R Dalebout H Holst S van Pelt G Farina-Sarasqueta A . N-glycomic signature of stage II colorectal cancer and its association with the tumor microenvironment. Mol Cell Proteomics (2021) 20:100057. doi: 10.1074/mcp.RA120.002215 33581319 PMC7973300

[2] Jia R Song L Fei Z Qin C Zhao Q . Long noncoding RNA ftx regulates the protein expression profile in HCT116 human colon cancer cells. Proteome Sci (2022) 20(1):7. doi: 10.1186/s12953-022-00187-1 35490216 PMC9055732

[3] Fanayan S Smith JT Lee LY Yan F Snyder M Hancock WS . Proteogenomic analysis of human colon carcinoma cell lines LIM1215, LIM1899, and LIM2405. J Proteome Res (2013) 12(4):1732–42. doi: 10.1021/pr3010869 23458625

[4] Rho JH Qin S Wang JY Roehrl MH . Proteomic expression analysis of surgical human colorectal cancer tissues: up-regulation of PSB7, PRDX1, and SRP9 and hypoxic adaptation in cancer. J Proteome Res (2008) 7(7):2959–72. doi: 10.1021/pr8000892 PMC2693877 18549262

[5] Balog CI Stavenhagen K Fung WL Koeleman CA McDonnell LA Verhoeven A . N-glycosylation of colorectal cancer tissues: a liquid chromatography and mass spectrometry-based investigation. Mol Cell Proteomics (2012) 11(9):571–85. doi: 10.1074/mcp.M111.011601 PMC3434767 22573871

[6] Kasprzak A . Insulin-like growth factor 1 (IGF-1) signaling in glucose metabolism in colorectal cancer. Int J Mol Sci (2021) 22(12):6434. doi: 10.3390/ijms22126434 PMC8234711 34208601

[7] Yang P Jiang Y Fischer SM . Prostaglandin E3 metabolism and cancer. Cancer Lett (2014) 348(1-2):1–11. doi: 10.1016/j.canlet.2014.03.010 24657656 PMC4366418

[8] Wang D DuBois RN . Role of prostanoids in gastrointestinal cancer. J Clin Invest. (2018) 128(7):2732–42. doi: 10.1172/JCI97953 PMC6026007 29733297

[9] Fang S Fang X . Advances in glucose metabolism research in colorectal cancer. BioMed Rep (2016) 5(3):289–95. doi: 10.3892/br.2016.719 PMC4998148 27602209

[10] Park B Kim JY Riffey OF Dowker-Key P Bruckbauer A McLoughlin J . Pyruvate kinase M1 regulates butyrate metabolism in cancerous colonocytes. Sci Rep (2022) 12(1):8771. doi: 10.1038/s41598-022-12827-9 35610475 PMC9130307

[11] Grennan AK . Protein s-nitrosylation: potential targets and roles in signal transduction. Plant Physiol (2007) 144(3):1237–9. doi: 10.1104/pp.104.900228 PMC1914129 17616506

[12] Williams JL Ji P Ouyang N Kopelovich L Rigas B . Protein nitration and nitrosylation by NO-donating aspirin in colon cancer cells: Relevance to its mechanism of action. Exp Cell Res (2011) 317(10):1359–67. doi: 10.1016/j.yexcr.2011.03.001 PMC3096692 21406194

[13] Chen YJ Ching WC Chen JS Lee TY Lu CT Chou HC . Decoding the s-nitrosoproteomic atlas in individualized human colorectal cancer tissues using a label-free quantitation strategy. J Proteome Res (2014) 13(11):4942–58. doi: 10.1021/pr5002675 25040305

[14] Leon-Bollotte L Subramaniam S Cauvard O Plenchette–Colas S Paul C Godard C . S-nitrosylation of the death receptor fas promotes fas ligand–mediated apoptosis in cancer cells. Gastroenterology (2011) 140(7):2009–18.e4. doi: 10.1053/j.gastro.2011.02.053 21354149

[15] Martinez-Ruiz A Lamas S . Detection and identification of s-nitrosylated proteins in endothelial cells. Methods Enzymol (2005) 396:131–9. doi: 10.1016/S0076-6879(05)96013-8 16291228

[16] Foster MW Forrester MT Stamler JS . A protein microarray-based analysis of s-nitrosylation. Proc Natl Acad Sci United States America. (2009) 106(45):18948–53. doi: 10.1073/pnas.0900729106 PMC2776442 19864628

[17] Kone BC . S-nitrosylation: Targets, controls and outcomes. Curr Genomics (2006) 7(5):301–10. doi: 10.2174/138920206778604340

[18] Derakhshan B Hao G Gross SS . Balancing reactivity against selectivity: the evolution of protein s-nitrosylation as an effector of cell signaling by nitric oxide. Cardiovasc Res (2007) 75(2):210–9. doi: 10.1016/j.cardiores.2007.04.023 PMC1994943 17524376

[19] Li K Huang M Xu P Wang M Ye S Wang Q . Microcystins-LR induced apoptosis *via* s-nitrosylation of GAPDH in colorectal cancer cells. Ecotoxicol Environ Saf (2020) 190:110096. doi: 10.1016/j.ecoenv.2019.110096 31901813

[20] Wang X Zhou W Gao Z Lv X . Mass spectrometry analysis of s-nitrosylation of proteins and its role in cancer, cardiovascular and neurodegenerative diseases. TrAC Trends Analytical Chem (2022) 152:116625. doi: 10.1016/j.trac.2022.116625

[21] Helmer RA Martinez-Zaguilan R Kaur G Smith LA Dufour JM Chilton BS . Helicase-like transcription factor-deletion from the tumor microenvironment in a cell line-derived xenograft model of colorectal cancer reprogrammed the human transcriptome-s-nitroso-proteome to promote inflammation and redirect metastasis. PloS One (2021) 16(5):e0251132. doi: 10.1371/journal.pone.0251132 34010296 PMC8133447

[22] Yang H Oh C-K Amal H Wishnok JS Lewis S Schahrer E . Mechanistic insight into female predominance in alzheimer's disease based on aberrant protein s-nitrosylation of C3. Sci Adv (2022) 8(50):eade0764–eade. doi: 10.1126/sciadv.ade0764 PMC9750152 36516243

[23] Lee H Shin N Song M Kang UB Yeom J Lee C . Analysis of nuclear high mobility group box 1 (HMGB1)-binding proteins in colon cancer cells: clustering with proteins involved in secretion and extranuclear function. J Proteome Res (2010) 9(9):4661–70. doi: 10.1021/pr100386r 20812762

[24] Torres S García-Palmero I Marín-Vicente C Bartolomé RA Calviño E Fernández-Aceñero MJ . Proteomic characterization of transcription and splicing factors associated with a metastatic phenotype in colorectal cancer. J Proteome Res (2018) 17(1):252–64. doi: 10.1021/acs.jproteome.7b00548 29131639

[25] Zheng P Liu YX Chen L Liu XH Xiao ZQ Zhao L . Stathmin, a new target of PRL-3 identified by proteomic methods, plays a key role in progression and metastasis of colorectal cancer. J Proteome Res (2010) 9(10):4897–905. doi: 10.1021/pr100712t 20806969

[26] Guo J Gaffrey MJ Su D Liu T Camp DG 2nd Smith RD . Resin-assisted enrichment of thiols as a general strategy for proteomic profiling of cysteine-based reversible modifications. Nat Protoc (2014) 9(1):64–75. doi: 10.1038/nprot.2013.161 24336471 PMC4038159

[27] Forrester MT Foster MW Benhar M Stamler JS . Detection of protein s-nitrosylation with the biotin-switch technique. Free Radic Biol Med (2009) 46(2):119–26. doi: 10.1016/j.freeradbiomed.2008.09.034 PMC3120222 18977293

[28] Fung KY Lewanowitsch T Henderson ST Priebe I Hoffmann P McColl SR . Proteomic analysis of butyrate effects and loss of butyrate sensitivity in HT29 colorectal cancer cells. J Proteome Res (2009) 8(3):1220–7. doi: 10.1021/pr8009929 19195990

[29] Huang JZ Chen M Chen D Gao XC Zhu S Huang H . A peptide encoded by a putative lncRNA HOXB-AS3 suppresses colon cancer growth. Mol Cell (2017) 68(1):171–84.e6. doi: 10.1016/j.molcel.2017.09.015 28985503

[30] Han P Chen C . Detergent-free biotin switch combined with liquid chromatography/tandem mass spectrometry in the analysis of s-nitrosylated proteins. Rapid Commun Mass Spectrom (2008) 22(8):1137–45. doi: 10.1002/rcm.3476 18335467

[31] Yang W Shi J Zhou Y Liu T Li J Hong F . Co-Expression network analysis identified key proteins in association with hepatic metastatic colorectal cancer. Proteomics Clin Appl (2019) 13(6):e1900017. doi: 10.1002/prca.201900017 31397080

[32] Li F Sonveaux P Rabbani ZN Liu S Yan B Huang Q . Regulation of HIF-1alpha stability through s-nitrosylation. Mol Cell (2007) 26(1):63–74. doi: 10.1016/j.molcel.2007.02.024 17434127 PMC2905600

[33] Sanhueza C Bennett JC Valenzuela-Valderrama M Contreras P Lobos-Gonzalez L Campos A . Caveolin-1-Mediated tumor suppression is linked to reduced HIF1alpha s-nitrosylation and transcriptional activity in hypoxia. Cancers (Basel) (2020) 12(9):2349. doi: 10.3390/cancers12092349 PMC7565942 32825247

[34] Yan JF Kim H Jeong SK Lee HJ Sethi MK Lee LY . Integrated proteomic and genomic analysis of gastric cancer patient tissues. J Proteome Res (2015) 14(12):4995–5006. doi: 10.1021/acs.jproteome.5b00827 26435392 PMC5706558

[35] Gu Y Tang S Wang Z Cai L Lian H Shen Y . A pan-cancer analysis of the prognostic and immunological role of beta-actin (ACTB) in human cancers. Bioengineered (2021) 12(1):6166–85. doi: 10.1080/21655979.2021.1973220 PMC8806805 34486492

[36] Krzystek-Korpacka M Diakowska D Bania J Gamian A . Expression stability of common housekeeping genes is differently affected by bowel inflammation and cancer: implications for finding suitable normalizers for inflammatory bowel disease studies. Inflammation Bowel Dis (2014) 20(7):1147–56. doi: 10.1097/MIB.0000000000000067 24859296

[37] Lange CPE Laird PW . Clinical applications of DNA methylation biomarkers in colorectal cancer. Epigenomics (2013) 5(2):105–8. doi: 10.2217/epi.13.4 23566085

[38] Huang CY Lee KC Tung SY Huang WS Teng CC Lee KF . 2D-DIGE-MS proteomics approaches for identification of gelsolin and peroxiredoxin 4 with lymph node metastasis in colorectal cancer. Cancers (Basel) (2022) 14(13):3189. doi: 10.3390/cancers14133189 PMC9265116 35804959

[39] Martinez-Aguilar J Molloy MP . Label-free selected reaction monitoring enables multiplexed quantitation of S100 protein isoforms in cancer cells. J Proteome Res (2013) 12(8):3679–88. doi: 10.1021/pr400251t 23782132

[40] Zhang X Ai F Li X She X Li N Tang A . Inflammation-induced S100A8 activates Id3 and promotes colorectal tumorigenesis. Int J Cancer. (2015) 137(12):2803–14. doi: 10.1002/ijc.29671 26135667

[41] Ichikawa M Williams R Wang L Vogl T Srikrishna G . S100A8/A9 activate key genes and pathways in colon tumor progression. Mol Cancer Res (2011) 9(2):133–48. doi: 10.1158/1541-7786.MCR-10-0394 PMC3078037 21228116

[42] Lim SY Yuzhalin AE Gordon-Weeks AN Muschel RJ . Tumor-infiltrating monocytes/macrophages promote tumor invasion and migration by upregulating S100A8 and S100A9 expression in cancer cells. Oncogene (2016) 35(44):5735–45. doi: 10.1038/onc.2016.107 PMC4961254 27086923

[43] Kuranaga Y Sugito N Shinohara H Tsujino T Taniguchi K Komura K . SRSF3, a splicer of the PKM gene, regulates cell growth and maintenance of cancer-specific energy metabolism in colon cancer cells. Int J Mol Sci (2018) 19(10):3012. doi: 10.3390/ijms19103012 PMC6213643 30279379

[44] Siragusa M Thole J Bibli SI Luck B Loot AE de Silva K . Nitric oxide maintains endothelial redox homeostasis through PKM2 inhibition. EMBO J (2019) 38(17):e100938. doi: 10.15252/embj.2018100938 31328803 PMC6717893

[45] Du ZX Wang HQ Zhang HY Gao DX . Involvement of glyceraldehyde-3-phosphate dehydrogenase in tumor necrosis factor-related apoptosis-inducing ligand-mediated death of thyroid cancer cells. Endocrinology (2007) 148(9):4352–61. doi: 10.1210/en.2006-1511 17540725

[46] Hu ZY Xiao L Bode AM Dong Z Cao Y . Glycolytic genes in cancer cells are more than glucose metabolic regulators. J Mol Med (Berl) (2014) 92(8):837–45. doi: 10.1007/s00109-014-1174-x 24906457

[47] Stanley CA Lieu YK Hsu BY Burlina AB Greenberg CR Hopwood NJ . Hyperinsulinism and hyperammonemia in infants with regulatory mutations of the glutamate dehydrogenase gene. New Engl J Med (1998) 338(19):1352–7. doi: 10.1056/NEJM199805073381904 9571255

[48] Ayiomamitis GD Notas G Vasilakaki T Tsavari A Vederaki S Theodosopoulos T . Understanding the interplay between COX-2 and hTERT in colorectal cancer using a multi-omics analysis. Cancers (Basel) (2019) 11(10):1536. doi: 10.3390/cancers11101536 PMC6827032 31614548

