# Supplementary figures and images for: Proteome profiling of endogenous and potential S-nitrosylation in colorectal cancer
Source: Front Endocrinol (Lausanne). 2023 Apr 14;14:1153719. doi: 10.3389/fendo.2023.1153719 (PMC10140627; doi:10.3389/fendo.2023.1153719)

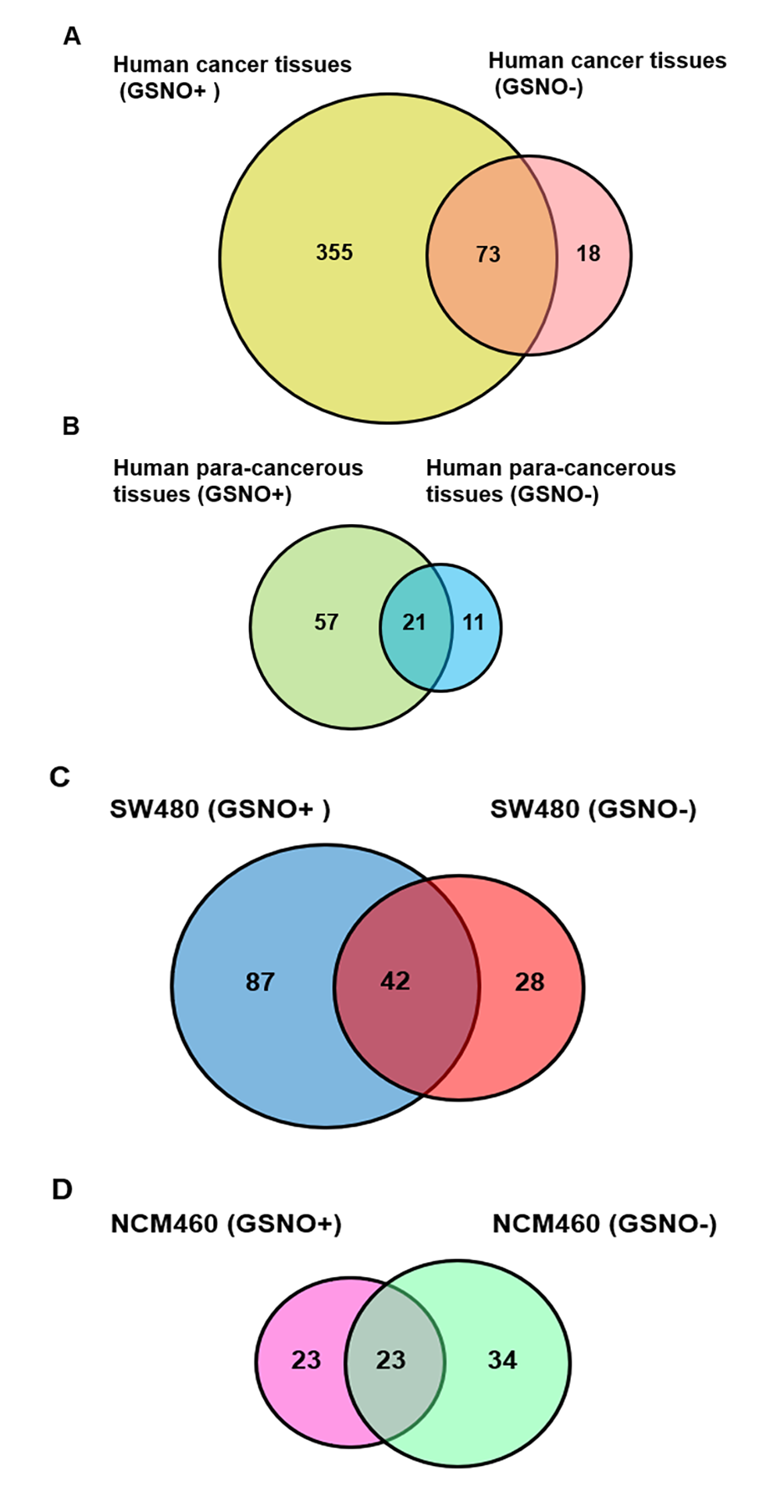

Supplement: Supplementary file 1 [file Image_1.tif]
